# Mapping Risk for Psychiatric Disorders to Brain Regions Using Spatial Transcriptomics

**DOI:** 10.1016/j.bpsgos.2026.100752

**Published:** 2026-06-01

**Authors:** Aodán Laighneach

**Affiliations:** Centre for Neuroimaging, Cognition and Genomics and Institute for Health Discovery and Innovation, University of Galway, Galway, Ireland

The spatial arrangement within a tissue encodes important information pertaining to the communication between cells, as well as the presence of nutrients and exposure to external stimuli (1). For brain tissue, this could represent, for example, neuronal connectivity and the relationship of neurons with nearby glial support cells. In some cases, such as when undertaking magnetic resonance imaging (MRI)-based investigations, it is possible to gain direct, high-precision spatial insights into brain structure or function, but these approaches do not infer molecular function. Techniques that do provide in-depth molecular data, such as traditional bulk RNA sequencing (RNA-seq) and, more recently, single-cell/nucleus RNA-seq, generally lack a precise spatial element. Spatial transcriptomics (ST), particularly sequencing-based ST, helps bridge this gap by combining high-throughput gene expression measurement and meaningful high-resolution positional information (2). These methods involve assaying the gene expression profiles of an array of many small, evenly spaced spots from a tissue capture area and using a combination of barcoded probes and high-resolution imaging taken before expression profiling to assign location.

The approach used in a recent study by Baran *et al.* (3), published in *Biological Psychiatry: Global Open Science,* leverages these ST data (4) and a contemporary statistical approach (5) to provide valuable insights into how common genetic liability for schizophrenia (SCZ), bipolar disorder (BD), and major depression (MDD) may be partitioned spatially within subfields of the human hippocampus. As described in Song *et al.* (5), this is accomplished by 1) calculating gene specificity for each ST spot; 2) assigning gene specificity to variants based on their location and epigenetic maps; 3) performing stratified linkage disequilibrium score regression (s-LDSC) based on genome-wide association study (GWAS) summary statistics; and 4) aggregating individual spot *p* values to reflect a per-region enrichment statistic. Baran *et al.* (3) identified enriched regions and used cellular deconvolution to annotate spots and investigate enrichments based on cell-type composition. Finally, the authors investigated whether the same hippocampal subfields are enriched for rare damaging coding variants for SCZ.

The hippocampus is a highly relevant region because it is crucial in cognitive processes and has been implicated as altered in these psychiatric disorders based on MRI and biomarker data. It is also divided into histologically distinguishable subfields [see Baran *et al.* (3), Figure 2], which lends itself well to spatial investigation. Baran *et al.* (3) began by testing for relative enrichments in genetic liability for SCZ, BD, and MDD within the subfields of the hippocampus. Of the 13 subfields, 6 (cornu ammonis [CA] area 1 [CA1], CA3, CA4, dentate gyrus [DG] granule cell layer, DG subgranular zone, and subiculum) showed enrichment for liability. The 2 most striking findings are related to some inconsistencies among these subfields: the relative lack of enrichment in MDD (although the authors pointed out that this may be related to lower overall heritability of MDD compared with SCZ or BD) and the divergence of liability for SCZ and BD in the DG subfields, where BD showed significantly stronger enrichments.

Given the strong overlap in genetic risk for SCZ and BD in terms of common variation (6), some level of divergence between the 2 disorders in the enrichment of liability within the DG represents a potentially important insight into their nonshared genetic basis. Being able to annotate these differences in terms of position is highly valuable. There is some evidence that the DG may be altered at the cellular level in BD. Induced pluripotent stem cells harvested from individuals with BD reprogrammed into granule cell-like neurons of the DG show aberrant electrophysiological properties, especially in the context of clinical responsiveness to the mood stabilizer lithium (7). This has led some to consider whether lithium’s possible effect on neurogenesis may be relevant (8). Baran *et al.* (3) followed up this finding by probing genes with the highest specificity for the DG subfields and investigating their association with SCZ and BD. Among the genes identified as being substantially more associated with BD was *SPTBN2* (DG granular cell layer specificity), a gene with structural roles associated with the glutamate synapse.

Although ST provides excellent positional information over a designated area, achieving this at individual cell-level resolution is difficult, and robust deconvolution approaches remain crucial for adding this level of information (1). Between SCZ and BD, Baran *et al.* (3) found highly consistent enrichment of cellular profiles, largely dominated by excitatory glutamatergic neurons and mixed populations of excitatory and vascular cells in enriched ST regions. Although not necessarily a novel finding, this supports previous non-ST approaches showing enrichments of liability within these cell types. Again, there is some divergence between SCZ and BD, with liability for BD shown to be enriched within genes specific to inhibitory (GABAergic [gamma-aminobutyric acidergic]) neurons of the subiculum, as is liability for MDD. The authors pointed out that the enrichment of both excitatory and inhibitory regions of the subiculum may be related to the subiculum’s role as the hippocampal output structure, through which it communicates with nearby brain regions, including those involved in limbic and reward circuitry.

Although common psychiatric disorders such as SCZ are often thought to be driven by common genetic liability (9), the biological insights obtained from studying rare and ultrarare risk variants are also valuable. Baran *et al.* (3) expanded on their common-variant findings by testing whether genes with high specificity for each subfield harbor an excess of rare damaging variants for SCZ. A number of subfields (CA4, subiculum, and the granular cell layer of the DG) showed convergence with results from common SCZ variation. The mechanisms by which common low-effect variants and rare higher-effect variants increase risk for SCZ are different. Therefore, enrichments within genes, pathways, cell types, and brain regions from a genetic perspective are inherently robust findings and indicative of a general intolerance to genetic changes. In SCZ, for example, genes related to glutamatergic function represent an important source of convergence between common and rare genetic risk (10). In general, these convergent associations are an excellent resource when considering animal and cell models (9), and are highly relevant in this context, where harvesting tissue comes with many impracticalities and potential confounds.

This work can be built upon in several ways. First, findings within the hippocampus show strong, consistent enrichments, with spatial arrangement proving to be important. Having the ability to assay ST across multiple brain regions with a similar or higher degree of resolution would help investigators compare the magnitude of enrichment within hippocampal subfields to other areas. Second, using a coprofiling approach with spatial epigenomics and spatial proteomics will facilitate an understanding of the mechanisms involved between genetic liability, gene regulation, gene expression, and biologically meaningful changes in this domain. Third, multi-ancestry and sex-specific effects were not investigated because of a limitation of s-LDSC and potential biases. However, larger non-European GWAS datasets and more flexible statistical approaches may facilitate this. Finally, the ST data used in this work (4) are from 10 neurotypical adult donors. However, if developmental ST data were used, it would be possible to follow the spatial enrichments in liability over a temporal window and gain insights into the neurodevelopmental nature of psychiatric disorders.

Overall, the work conducted by Baran *et al.* (3) highlights the importance of the effective combination of high-quality ST datasets and the use of novel statistical techniques to make progress in understanding the biology of psychiatric disorders. The authors contribute novel insights into how liability for SCZ, BD, and MDD may be distributed in the hippocampus and which aspects may represent shared or unique signatures of individual disorders, which is notably a clear importance of the DG in BD. Findings were robust and consistent, especially those implicating glutamatergic systems. The authors emphasize the utility of ST and cellular deconvolution and demonstrate a workflow that can be applied to a wide range of genetic disorders and ST datasets.
